# Simulation Study of Enhancement-Mode *β*-Ga_2_O_3_ MOSFETs on a Novel P-Ga_2_O_3_/AlN/SiC Substrate

**DOI:** 10.3390/mi17050595

**Published:** 2026-05-13

**Authors:** Wenhai Lu, Chunyu Zhou, Danying Wang, Yong Liu, Peiyi Wang, Guanyu Wang

**Affiliations:** 1 State Key Laboratory of Metastable Materials Science and Technology and Hebei Key Laboratory of Microstructural Material Physics, School of Science Yanshan University, Qinhuangdao 066004, China; luwenhai@stumail.ysu.edu.cn (W.L.); wangdanying@stumail.ysu.edu.cn (D.W.); yongliu@ysu.edu.cn (Y.L.); peiyi0428@stumail.ysu.edu.cn (P.W.); 2 School of Integrated Circuits, Chongqing University of Posts and Telecommunication, Chongqing 400065, China; wangguanyu@cqupt.edu.cn

**Keywords:** Ga_2_O_3_, peak temperature, breakdown voltages, threshold voltage, specific on-resistance

## Abstract

This work presents the design of a β-Ga_2_O_3_ MOSFET incorporating a P-type Ga_2_O_3_ buffer layer on a high-thermal-conductivity AlN/SiC composite substrate. The electrical characteristics of the device were simulated using Sentaurus TCAD. Results demonstrate that the integration of the composite substrate effectively mitigates self-heating effects, reducing the peak temperature ($T_{\max}$) from 776.5 K to 570.9 K at 300 K, while simultaneously increasing the threshold voltage ($V_{\mathrm{th}}$) from −0.35 V to 1.52 V. Through systematic optimization of the P-Ga_2_O_3_ buffer layer thickness and doping concentration, the device achieves a breakdown voltage ($V_{\mathrm{br}}$) of 4781 V, a power figure of merit (PFOM) of 2.18 GW/cm^2^, an $I_{DS}$, on/off ratio of 9.20 × 10^9^, and cut-off/maximum oscillation frequencies ($f_{t}$/$f_{\max}$) of 1.29 GHz and 1.40 GHz, respectively. These findings provide a theoretical foundation for developing β-Ga_2_O_3_-based power devices with high breakdown voltage, improved thermal conductivity, and low specific on-resistance ($R_{\mathrm{on},\mathrm{sp}}$).

## 1. Introduction

β-Ga_2_O_3_, as an emerging ultra-wide bandgap semiconductor material, possesses a wide bandgap of 4.8 eV, an extremely high critical breakdown field strength of 8 MV/cm, and a Baliga’s figure of merit (BFOM) of 3444 (Ω·cm^2^)·V^−2^ [1,2]. It can be grown via the melt method, which enables low-cost crystal production and shows promising potential for applications in high-voltage, high-power, high-efficiency, and compact electronic devices [3,4,5,6,7]. Recent studies on the β-Ga_2_O_3_ Metal-Oxide-Semiconductor Field-Effect Transistor (MOSFET) device have demonstrated breakdown voltages ($V_{br}$) exceeding 3 KV and a drain current ($I_{DS}$) on/off ratio greater than 10^10^, positioning these devices as promising candidates for high-voltage and high-power electronic applications [8,9]. β-Ga_2_O_3_ MOSFETs are classified into lateral and vertical configurations based on current transport orientation. Lateral β-Ga_2_O_3_ MOSFETs are categorized into semi-insulating Ga_2_O_3_ substrate configurations, which employ Fe/Mg-doped p-type materials, and hetero-integrated substrates utilizing high-thermal-conductivity materials such as SiC and Si to enhance thermal performance [4,9,10,11,12,13,14,15,16,17,18,19,20,21,22,23,24,25,26,27]. However, the enhancement of device $V_{br}$ is typically realized by increasing drift region length or reducing drift region doping concentration, though these approaches inherently elevate the specific on-resistance ($R_{on,sp}$), thereby constraining device applicability in high-voltage high-power systems.

Vertical β-Ga_2_O_3_ MOSFETs employing fin-shaped channels (FinFET) with top-side source electrodes and backside drain contacts achieve vertical current transport through drift regions with thickness decoupled from lateral dimensions [28,29]. This configuration enables electric field peak confinement within the bulk material through multi-gate structures while maintaining current pathways away from surface interfaces, thereby mitigating surface-state-induced reliability degradation and air-breakdown phenomena, which resolves the lateral device trade-off between conduction resistance and breakdown performance. In comparison with other wide-bandgap semiconductor materials, β-Ga_2_O_3_ exhibits relatively low thermal conductivity (10–30 W/m·K) [3,4,30], which results in significant self-heating effects (SHEs) during high-power operation [31,32,33]. Elevated temperatures intensify lattice scattering (phonon scattering), reducing electron mobility and inducing current collapse in the device’s saturation region phenomenon termed the SHE. This SHE also restricts the device’s development and application in high-power and large-current fields [4,34,35].

The utilization of 4H-SiC substrates with thermal conductivity of 280 W/m·K (measured via laser flash analysis at 300 K) in β-Ga_2_O_3_ devices significantly mitigates SHE [4,12,32,36]. However, the lattice mismatch between 4H-SiC and β-Ga_2_O_3_ during heteroepitaxial growth, thereby degrading device electrical performance [5,37]. To address these challenges, p-type β-Ga_2_O_3_ [11,38,39,40,41]/AlN/SiC heterostructure architecture is employed, where AlN serves as an epitaxial buffer layer due to its high thermal conductivity (260 W/m·K) and wide bandgap (6.2 eV) [42]. This combination enhances current drive capability by reducing interface scattering and improving heat dissipation [4,43]. The incorporation of P-type β-Ga_2_O_3_ modulates electric field distribution in the channel region through hole-induced charge compensation, thereby enhancing the $V_{br}$ while simultaneously reducing the $R_{on,sp}$ [43,44,45,46,47]. By optimizing the thickness and doping concentration of the p-type β-Ga_2_O_3_ buffer layer and the 4H-SiC substrate, two enhanced device structures were finally designed, namely GaOPSiC1 with the best thermal conductivity and GaOPSiC2 with the highest breakdown characteristics. Compared with the existing reported device structures, they have significant advantages. The breakdown voltage can reach up to 4781 V, and the power quality factor is 2.18 GW/cm^2^. This device provides a theoretical basis for the development of high-voltage and high-thermal conductivity gallium oxide devices.

## 2. Device Structure

**Figure 1:**
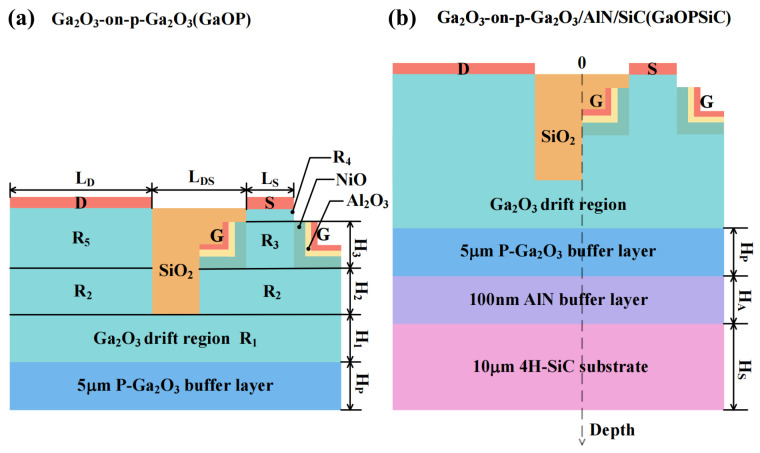
Schematic diagrams of (**a**) GaOP (**b**) GaOPSiC device structures.

The device proposed in this study is depicted in Figure 1. Figure 1a presents the GaOP (β-Ga_2_O_3_-on-P-Ga_2_O_3_) structure, which employs an iron or magnesium-doped p-type β-Ga_2_O_3_ substrate with a doping concentration of $N_{p}$ = 2 × 10^10^–2 × 10^20^ cm^−3^ and thickness of $H_{p}$ = 1–10 μm [11,38,39]. Above the substrate, N-type β-Ga_2_O_3_ drift region is positioned, which is partitioned into five segments labeled $R_{1}$–$R_{5}$ with doping concentrations of $N_{1}$–$N_{5}$ ($N_{1}$ = $N_{2}$ = 1.45 × 10^16^ cm^−3^, $N_{3}$ = 2 × 10^15^ cm^−3^, $N_{4}$ = 5 × 10^19^ cm^−3^, $N_{5}$ = 2 × 10^18^ cm^−3^) and thicknesses of $H_{1}$–$H_{5}$ ($H_{1}$ = 4 μm, $H_{2}$ = 5 μm, $H_{3}$ = 0.80 μm, $H_{4}$ = 0.11 μm, $H_{5}$ = 0.91 μm). $L_{S}$ ($L_{S}$ = 0.22 μm) and $L_{D}$ ($L_{D}$ = 20.20 μm) are the source/drain electrode lengths. $L_{DS}$ ($L_{DS}$ = 11 μm) defines the spacing between source and drain regions, with SiO_2_ as the isolation layer. The gate dielectric employs a composite structure of nickel oxide and Al_2_O_3_ to enhance gate modulation of the channel and suppress gate leakage current [22]. The nickel oxide layer is boron-doped with a concentration of 2 × 10^17^ cm^−3^, while the Al_2_O_3_ layer remains intrinsic. Their thicknesses are 0.1 μm and 0.03 μm, respectively. The gate electrode employs a platinum layer with a thickness of 0.03 μm, featuring a metal work function ($\phi_{M}$) of 6.30 eV. Figure 1b illustrates the GaOPSiC(β-Ga_2_O_3_-on-P-Ga_2_O_3_/AlN/SiC) configuration. Compared to Figure 1a, the adoption of a P-type β-Ga_2_O_3_/AlN/SiC composite substrate structure enhances the device’s thermal performance. The AlN buffer layer has a thickness of $H_{A}$ = 0.10 μm, while the 4H-SiC substrate exhibits a thickness of $H_{S}$ = 10–18 μm and a doping concentration of $N_{S}$ = 5 × 10^13^ cm^−3^. Except for the dimensions and doping concentrations marked in Figures 4–6, no other changes are made to the device.

The physical model is an indispensable part of the numerical solution process [48,49]. The physical models used in this paper mainly include the Bennett–Wilson model [50], which is employed to describe the energy band structure of semiconductors. The Philips unified mobility model proposed by Krishnaswamy [51] is used to illustrate the effects of temperature, lattice scattering, and electric field on mobility. The ionized impurity scattering Arora model [52] and the generation-combination SRH model [53] for describing the process of carriers exchanging between the conduction band and the valence band of impurities, and the avalanche breakdown Avalanche model [54] and the thermodynamic model [55] are used.

## 3. Results and Discussion

**Figure 2:**
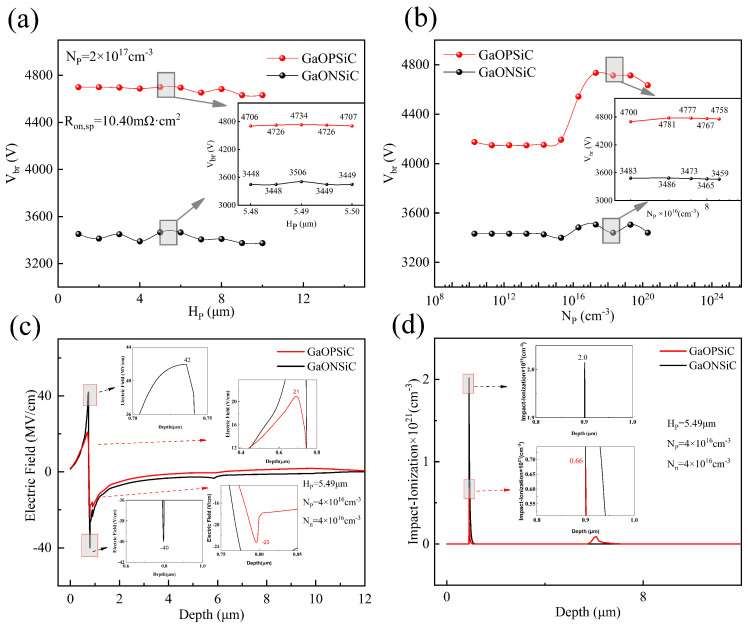
Breakdown characteristic simulation results of GaOPSiC and GaONSiC devices. (**a**) $V_{br}$ variation with $H_{p}$ at $N_{p}$ = 2 × 10^17^ cm^−3^. (**b**) Breakdown voltage variation with $N_{p}$ at $H_{p}$ = 5.49 μm. (**c**) Electric field profile along the vertical depth beneath the left gate electrode. (**d**) Impact ionization rate distribution along vertical depth.

**Figure 3:**
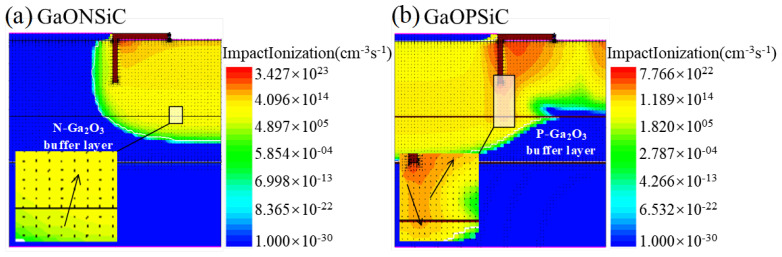
The distribution images of the internal electric field and the collision ionization rate during the GaONSiCand GaOPSiC device breakdown.

**Figure 4:**
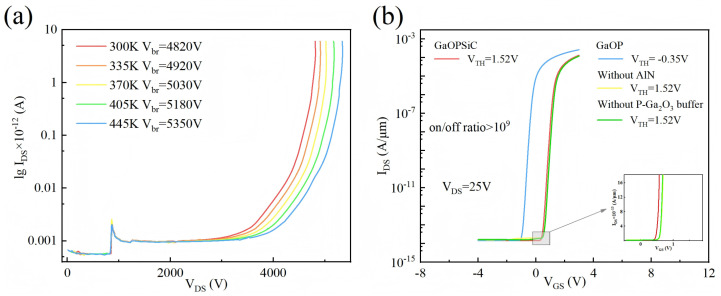
Device simulation results: (**a**) $V_{br}$ analysis *$I_{DS}$-$V_{DS}$* for GaOPSiC accounting for SHE at $H_{p}$ = 5.49 μm and $N_{p}$ = 2 × 10^17^ cm^−3^; (**b**) *$I_{DS}$-$V_{GS}$* in log scale of GaOPSiC, GaOP, without AlN, and without P-Ga_2_O_3_ buffer.

**Figure 5:**
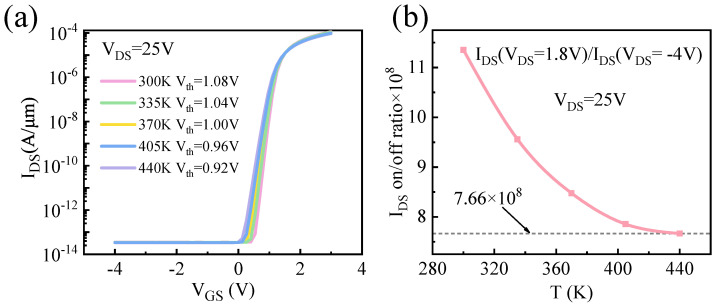
Simulation presents SHE in GaOPSiC with $V_{DS}$ = 25 V: (**a**) *$I_{DS}$-$V_{GS}$* in log scale; (**b**) $I_{DS}$ on/off ratio vs. *T*.

**Figure 6:**
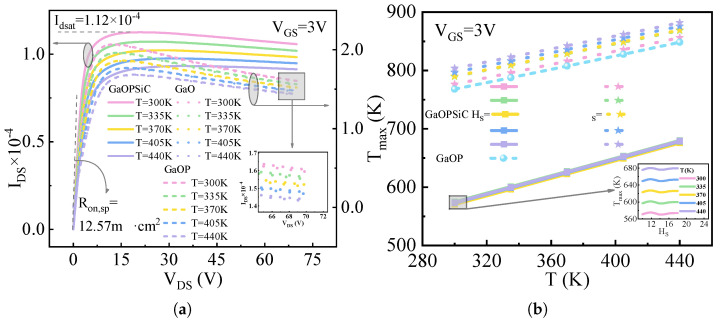
Simulation of self-heating effect in device: (**a**) $I_{DS}$-$V_{DS}$ characteristics at $V_{GS}$ = 3 V and $H_{S}$ = 10 μm; (**b**) $T_{max}$ vs. *T* for different $H_{S}$ at $V_{GS}$ = 3 V.

**Figure 7:**
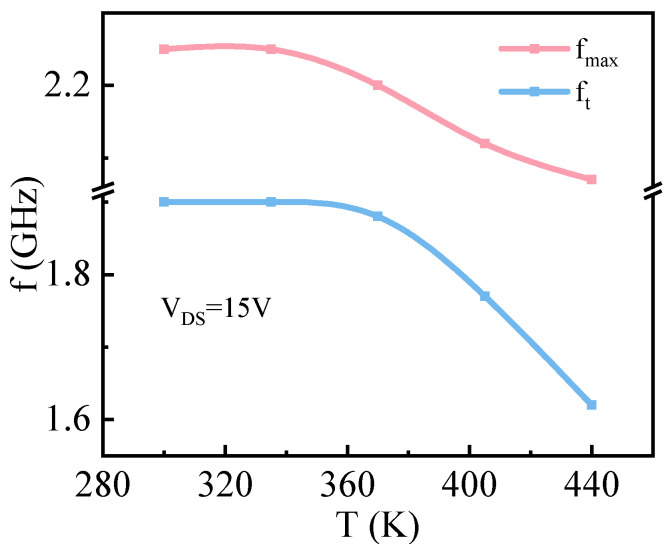
*f* vs. *T* of GaOPSiC at $V_{DS}$ = 15 V.

The simulation of this device was carried out using the TCAD software version of 2018. with the results documented in Figure 2, Figure 3, Figure 4, Figure 5, Figure 6 and Figure 7. Figure 2 presents a comparison of simulation results between GaONSiC and GaOPSiC structures designed on an N-type β-Ga_2_O_3_/AlN/SiC substrate stack, where (a) illustrates the $V_{br}$ variation with $H_{p}$ at $N_{p}$ = 2 × 10^17^ cm^−3^, and (b) shows the $V_{br}$ dependence on $N_{p}$ at $H_{p}$ = 5.49 μm. Here, the breakdown voltage is defined as the value of the drain-source voltage $V_{DS}$ when the drain current $I_{DS}$ = 1 × 10^−11^ A.

The simulation results demonstrate that GaOPSiC exhibits superior breakdown capability compared to GaONSiC, with GaOPSiC achieving record-high $V_{br}$ of 4781 V at an optimized doping concentration ($N_{p}$ = 4 × 10^16^ cm^−3^) and structural thickness ($H_{p}$ = 5.49 μm). This operational point corresponds to $R_{on,sp}$ of 10.40 mΩ·cm^2^ and an exceptional power figure of merit (PFOM) of 2.20 GW·cm^−2^, underscoring its potential for high-power applications. In comparison with the GaONSiC of identical dimensions, GaOPSiC presents 1295 V enhancement in $V_{br}$, 0.55 mΩ·cm^2^ reduction in $R_{on,sp}$, and 1.10 GW·cm^−2^ improvement in PFOM.

Figure 3 shows the distribution of electric field and collision ionization rate at various locations during the breakdown of GaOPSiC and GaONSiC devices. The arrows indicate the direction of the electric field in the Ga_2_O_3_ buffer layer. It can be seen that the collision ionization rate is the highest in the drift region at the lower left of the channel, so this area has the densest electron distribution and is the breakdown point. The electric field of GaONSiC devices points towards P-NiO, while for GaOPSiC devices, the drift region’s electric field points towards P-NiO and a part of it also points towards the P-Ga_2_O_3_ buffer layer. Figure 2c,d present the vertical electric field strength distribution profiles and impact ionization rate variation profiles for the two devices. Compared to the GaONSiC structure, under identical $H_{p}$ and $N_{p}$ conditions, the GaOPSiC structure exhibits a lower electric field and collision ionization rate. The integration of the P-Ga_2_O_3_ buffer layer in the GaOPSiC device induces the formation of the P/N/P heterostructure, which effectively reduces the electric field peak intensity and collision ionization rate in the channel region. This structural modification consequently elevates the $V_{br}$, decreases the $R_{on,sp}$, and thereby enhances the PFOM.

Figure 4 presents the device simulation results, where Figure 4a presents the breakdown characteristics of GaOPSiC devices simulated over a temperature range of 300–440 K (with $H_{p}$ = 5.49 μm and $N_{p}$ = 2 × 10^17^ cm^−3^), explicitly accounting for SHE. This occurs because between 300 and 440 K, lattice scattering dominates carrier mobility [31,56]. Elevated temperatures intensify β-Ga_2_O_3_ lattice vibrations, increase polar optical phonon concentration, and enhance scattering probability. The resulting mobility degradation raises the saturation breakdown field, ultimately increasing $V_{br}$.

Figure 4b presents the transfer characteristics of GaOPSiC, GaOP, without AlN (without AlN buffer layer compared with GaOPSiC), and without P-Ga_2_O_3_ buffer (without P-type β-Ga_2_O_3_ buffer layer compared with GaOPSiC, $N_{p}$ = 4 × 10^16^ cm^−3^) at $N_{p}$ = 4 × 10^16^ cm^−3^. The $V_{th}$ of without AlN, without P-Ga_2_O_3_ buffer, and GaOPSiC devices remain constant at 1.52 V under fixed $V_{DS}$ conditions, while the GaOP device exhibits a distinct threshold voltage of −0.35 V. The 4H-SiC substrate exerts the most significant influence on the $V_{th}$ of the device. The $V_{th}$ model of GaOPSiC devices is [57,58,59]

(1) $$V_{th}=\phi_{MS}-qN_{D}\left(\frac{L_{S}^{2}}{2\varepsilon_{0}\varepsilon_{D}}-\frac{L_{S}}{C_{ox}}\right)A$$

where $\phi_{MS}$ is the work function difference between the gate metal and the semiconductor, and *A* is the correction factor. When the substrate is 4H-SiC, the $\phi_{MS}$ values for the GaOP 4H-SiC, without AlN, and without P-Ga_2_O_3_ buffer configurations increase by 1.86 V compared to the GaOP structure. According to Equation (1), this leads to a corresponding increase in $V_{th}$ of 1.86 V. As shown in Figure 4, the $V_{th}$ exhibits a shift of 1.87 V, resulting in an error of 0.54% compared to the theoretical value, thereby validating the high accuracy and reliability of the model in predicting the device’s electrical characteristics.

Figure 5 presents simulation results for GaOPSiC devices accounting for SHE. Figure 5a illustrates transfer characteristic curves at temperatures ranging from 300 K to 440 K ($V_{DS}$ = 25 V), while Figure 5b presents the temperature dependence of the $I_{DS}$ on/off current ratio. The results indicate that as temperature increases, the $V_{th}$ decreases, accompanied by a corresponding reduction in the $I_{DS}$ on/off ratio. Because the elevated temperature reduces electron confinement in the p-type doped β-Ga_2_O_3_ buffer layer, it weakens channel electron restriction and increases carrier concentration. This sequential mechanism results in a reduced V_*th*_ and $I_{DS}$ on/off ratio. Notably, the device maintains a superior switching ratio of 7.66 × 10^8^ at 440 K, outperforming reported benchmarks: 9 × 10^6^ (GaOISi at 400 K [23]), 1.15 × 10^7^ (GaOISiC at 400 K [23]), and the previous maximum of 1 × 10^6^ at 423 K [9].

Figure 6 presents the simulation of the self-heating effect in the device, where Figure 6a presents the output characteristic curve of GaOPSiC, GaOP, and GaO (β-Ga_2_O_3_-on-P-Ga_2_O_3_-on-N-Ga_2_O_3_) devices under specified operating conditions: temperature range 300–440 K, $V_{GS}$ = 3 V, and $H_{S}$ = 10 μm. Saturation behavior is observed at a drain-source voltage of $V_{DS}$ = 15 V, with the corresponding saturated $I_{dsat}$ values presented as [60]

(2) $$I_{dsat}=\frac{1}{2}\frac{L_{S}}{H_{3}}\mu_{n}C_{ox}{\left(V_{GS}-V_{th}\right)}^{2}$$

where $H_{3}$ is the channel length, $L_{S}$ is the channel width, $\mu_{n}$ is the mobility of charge carriers, and $C_{ox}$ is the capacitance of gate oxide. It can be seen that with the increase in temperature, the drain saturation current ($I_{dsat}$) of the device decreases, the $R_{on,sp}$ increases, the current in the saturation region is attenuated, and the negative differential resistance appears. The decay rate of the current, Δ*I*, as a function of temperature is shown in Table 1, where Δ*I* is denoted by

(3) $$\Delta I=\frac{I_{dsat}-I_{ds}\left(V_{DS}=70V\right)}{I_{dsat}}\times 100\%$$

The Δ*I* exhibits a decreasing trend with increasing temperature, following the order of Δ*I*(GaO) > Δ*I*(GaOP) > Δ*I*(GaOPSiC). Figure 6b presents the temperature-dependent evolution of peak temperature ($T_{max}$) within GaOPSiC, GaOP, and GaO devices across the temperature range of 300 K to 440 K, demonstrating $T_{max}$ dependence on both ambient temperature and $H_{s}$. $T_{max}$ denotes the maximum internal temperature of the device, with its value being positively correlated with increased thermal resistance and more pronounced SHE [61,62]. As temperature increases, the $T_{max}$ of the device increases, with $T_{max}$ following the order: $T_{max}$(GaO) > $T_{max}$(GaOP) > $T_{max}$(GaOPSiC). For the GaO structure, $T_{max}$ decreases as the thickness of the β-Ga_2_O_3_ substrate increases. While $T_{max}$ decreases monotonically with Ga_2_O_3_ substrate thickness in GaO, it shows non-monotonic increase–decrease–increase dependence on 4H-SiC thickness in GaOPSiC with turning points at 12 μm and 14 μm.

As derived from Equation (2), elevated temperatures reduce carrier mobility, leading to a decrease in I_*dsat*_ and an increase in $R_{on,sp}$, with this mobility reduction also causing current degradation in the saturation region. Due to the significantly lower thermal conductivity of β-Ga_2_O_3_ compared to 4H-SiC, its utilization as a substrate leads to particularly severe thermal management challenges for the device. Under constant gate bias conditions, Equation (2) presents that for GaO and GaOP structures (with substrate thickness approximated as zero), increased substrate thickness leads to longer thermal dissipation pathways, elevated thermal resistance, and consequently greater $T_{max}$ and Δ*I*. This results in Δ*I*(GaO) exceeding Δ*I*(GaOP) and $T_{max}$(GaO) surpassing $T_{max}$(GaOP). The thermal resistance exhibits direct proportionality to the thermal dissipation path length and an inverse proportionality to the material’s thermal conductivity. Consequently, while thickening the high-thermal-conductivity 4H-SiC substrate in GaOPSiC devices elevates thermal resistance, it simultaneously enhances the structure’s effective thermal conductivity. This dual effect results in larger Δ*I* and higher $T_{max}$ for GaOP compared to GaOPSiC, as expressed by Δ*I*(GaOP) > Δ*I*(GaOPSiC), $T_{max}$(GaOP) > $T_{max}$(GaOPSiC). Within the $H_{S}$ ranges of 10–12 μm and 14–18 μm, increasing $H_{S}$ leads to elevated thermal resistance and reduced $T_{max}$ in the device. When $H_{S}$ operates between 12 and 14 μm, the rise in the $H_{S}$ value causes decreased the overall thermal conductivity and subsequent $T_{max}$ reduction. Notably, at $H_{S}$ = 14 μm, the device achieves its minimum $T_{max}$, indicating optimal thermal performance. The high thermal conductivity of the AlN/SiC substrate facilitates timely dissipation of internal heat, thereby suppressing the exacerbation of the SHE and mitigating temperature-induced power dissipation in the device. In heteroepitaxial growth, the interface layer formed during the early stages of growth typically exhibits poor crystal quality, resulting in a thermal conductivity lower than that of the bulk material [63]. During the manufacturing process, strict control of growth conditions—such as using high-purity N_2_ as the carrier gas, setting the growth temperature to 800–900 °C, maintaining the pressure at 10 kPa, and controlling the epitaxial growth rate to 4–7 nm/min—defects in materials such as Ga_2_O_3_ can be effectively reduced [64]. This enables heterogeneous integration with high-thermal-conductivity 4H-SiC substrates, thereby enhancing the material’s thermal conductivity.

Figure 7 shows the simulation curve of the frequency characteristic of GaOPSiC devices considering SHE at $V_{DS}$ = 15 V and temperatures ranging from 300 K to 440 K. As temperature increases, both the cut-off frequency (*f*_*t*_) and maximum operating frequency (*f*_*max*_) exhibit decreasing trends. This phenomenon can be attributed to the direct proportionality between $f_{t}/f_{max}$ and $g_{m}$, as derived from Equation (2) and the definition of $g_{m}$ [65,66].

(4) $$g_{m}=\frac{\partial I_{dsat}}{\partial V_{GS}}=\frac{Z}{L}\mu_{n}C_{ox}V_{dsat}$$

As the temperature rises, the mobility of electrons decreases, which in turn leads to a reduction in $g_{m}$, thereby causing the values of $f_{t}$ and $f_{max}$ to decrease as well.

By optimizing the thickness and doping concentration of the P-Ga_2_O_3_ buffer layer and the 4H-SiC substrate, two device structures were obtained: GaOPSiC1 and GaOPSiC2. Among these, GaOPSiC1 has $N_{p}$ = 4 × 10^16^ cm^−3^, and GaOPSiC1 has $N_{p}$ = 4 × 10^16^ cm^−3^, $H_{p}$ = 5.49 μm, and $H_{S}$ = 11 μm, exhibiting the lowest Δ*I* and best thermal conductivity, while GaOPSiC2 has $N_{p}$ = 4 × 10^16^ cm^−3^, $H_{p}$ = 5.49 μm, and $H_{S}$ = 10 μm, with the highest breakdown voltage and PFOM. The performance parameters of the two devices are shown in Table 2.

**Figure 8:**
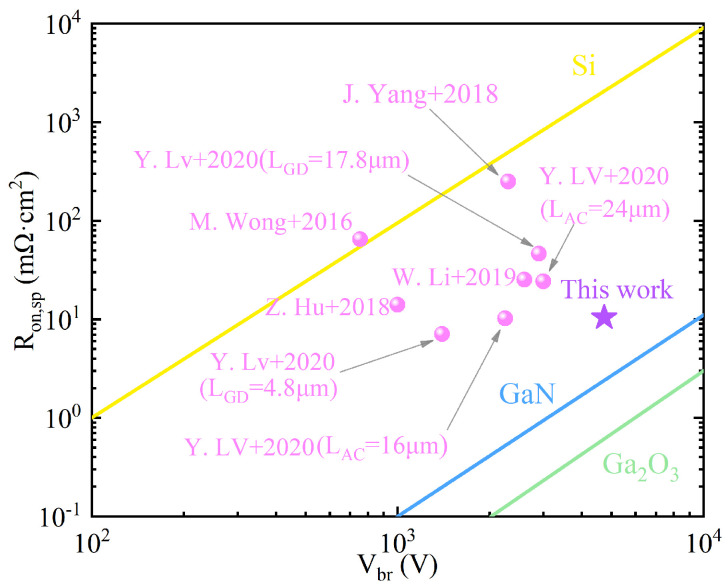
Comparison of specific on-resistance ($R_{on,sp}$) and breakdown voltage ($V_{br}$) between the device designed in this work (simulation results) and those of previously reported gallium oxide MOSFETs (experimental results) [11,18,20,67,68,69].

The breakdown voltage and the ratio of on-resistance are important parameters for evaluating the power quality factor of a device. To demonstrate the excellent performance of this structure, by referring to the literature, the experimental data of some published Ga_2_O_3_ MOSFET devices with Si or SiC heterojunction substrates were compared with the results of this structure. The results are shown in Figure 8. It can be seen that the breakdown voltage and the power quality factor of this structure are both relatively large, and it has broad application prospects in the high-voltage and high-power fields.

## 4. Conclusions

In this work, the hetero-integration of β-Ga_2_O_3_ MOSFETs with a P-Ga_2_O_3_ buffer layer on AlN/SiC substrates is comprehensively investigated through TCAD simulations. By incorporating the p-type β-Ga_2_O_3_ buffer layer, the device achieved $V_{br}$ of 4781 V, $R_{on,sp}$ of 10.40 mΩ·cm^2^, and PFOM of 2.20 GW/cm^2^. The utilization of AlN/SiC substrates significantly improved thermal management, reducing $T_{max}$ from 776.50 K to 570.90 K under ambient conditions of 300 K. Key performance metrics included $V_{th}$ of 1.52 V and an $I_{DS}$ on/off ratio exceeding 9.20 × 10^9^. These advances enabled stable operation under high-voltage and high-temperature conditions, demonstrating substantial application potential in modern power electronic systems.

## Figures and Tables

**Table 1:** Variation in Δ*I* with temperature for GaOPSiC, GaOP, and GaO at temperatures of 300–440 K.

Crystal Planes	300 K	335 K	370 K	405 K	440 K
GaOPSiC	6.00%	4.92%	3.84%	2.92%	2.00%
GaO	22.8%	20.92%	18.82%	16.48%	14.68%
GaOP	22.42%	20.42%	18.42%	16.54%	14.66%

**Table 2:** Performance parameters of GaOPSiC1 and GaOPSiC2.

Device	$V_{\mathrm{th}}$ (V)	$I_{\mathrm{DS}}$ On/Off Ratio $\times \;10^{9}$	$R_{\mathrm{on},\mathrm{sp}}$ $\left(m\Omega \cdot {\mathrm{cm}}^{2}\right)$	PFOM $\left(\mathrm{GW}\cdot {\mathrm{cm}}^{-2}\right)$	$f_{t}$ (GHz)	$f_{\max}$ (GHz)	$V_{\mathrm{br}}$ (V)
GaOPSiC1	1.29	9.20	10.40	2.16	1.45	1.50	4738
GaOPSiC2	1.52	9.20	10.40	2.20	1.29	1.40	4781

## Data Availability

All reasonable requests for materials should be directed to and will be fulfilled by the corresponding author.
